# CCN1 stimulated the osteoblasts via PTEN/AKT/GSK3*β*/cyclinD1 signal pathway in Myeloma Bone Disease

**DOI:** 10.1002/cam4.2608

**Published:** 2019-11-26

**Authors:** Siyang Yan, Hui Liu, Zhaoyun Liu, Fengping Peng, Fengjuan Jiang, Lijuan Li, Rong Fu

**Affiliations:** ^1^ Department of Hematology Tianjin Medical University General Hospital Tianjin P.R. China; ^2^ Tianjin Medical University Tianjin P.R. China

**Keywords:** CCN1, GSK3*β*, hematological cancer, myeloma bone disease, osteoblast, stimulation

## Abstract

**Backgrounds:**

Myeloma‐related bone disease (MBD) is a common complication of multiple myeloma (MM), which can both decrease life quality and influence the prognosis of the patients. We have found that CCN1 stimulated proliferation and differentiation of osteoblasts in MM in vitro and in vivo, while its mechanism still remains unknown.

**Method:**

Bone marrow mononuclear cells were collected from MM patients and differentiated into the osteoblasts. After co‐culture with CCN1 in vitro, the intracellular signaling antibody array and western blot were performed to explore the signaling pathway. Furthermore, GSK3*β* inhibitor TWS119 was used to check the pathway of CCN1 might have on osteoblasts in vitro.

**Results:**

For the protein array kit, the expressions of GSK3*β*, 4E‐BP1, and PTEN are decreased in CCN1 group. For western blots, the CCN1 group also has lower expression comparing to the control group in PTEN (*P* = .031). Meanwhile p‐AKT and cyclinD1 levels have increased in the CCN1 group (*P* = .002, *P* = .039). After adding TWS119 as another group, western blot was performed again to verify the pathway. For upstream proteins PTEN and p‐AKT, TWS119 group has higher expression level compared to that in CCN1 group (*P* = .003, *P* = .001). And for downstream protein cyclinD1, TWS119 group also presented higher level than the control group (*P* = .02). CCN1 could have almost the same effect on GSK3*β* as the specific inhibitor TWS119 had.

**Conclusions:**

CCN1 can stimulate osteoblasts through PTEN/AKT/GSK3*β*/cyclinD1 pathway in MBD, which has the potential to be a novel therapy of MBD.

## Graphical abstract

CCN1 and GSK3*β* inhibitor TWS119 had the same effect on decreasing the viability of GSK3*β*. Control group was cultured only with medium, CCN1 group was cultured with CCN1 at concentration of 30 ng/mL and TWS119 group was cultured with TWS119 at concentration of 2 μmol/L. The three groups were all incubated for 72 hours. GAPDH and *β*‐actin served as loading control. *P < .05; **P < .01.

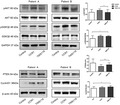

## 1 INTRODUCTION

Multiple myeloma is the second most common malignant hematologic disease with a yearly mortality rate of 4.1/100 000. It usually affects more old patients, with a median age at diagnosis of 65‐70 years.^1^, ^2^ Myeloma is also the most frequent cancer that can involve the skeleton. Over 80% of its patients have different degrees of damage on their bones, which is called the myeloma‐related bone disease (MBD).^3^, ^4^ Severe bone pain, osteolytic lesions, pathologic fractures, vertebral collapse, and hypercalcemia are the common symptoms for MBD.^5^ Comparing to other myeloma patients without MBD, the myeloma patients with pathological fractures would have 20% higher mortality and cost 50 000 dollars more for the care.^3^, ^6^, ^7^ As a characteristic complication, MBD can not only decrease the patients’ life quality but also have negative influence on the progression of myeloma. According to the previous research, the severity of the bone disease even correlates with tumor burden and myeloma prognosis.^5^, ^8^, ^9^ Thus, solving this problem can make great contribution to large amount of myeloma patients. Under physiological circumstance, the bone homeostasis is maintained by the co‐work of bone formation and bone resorption. And these two procedures are respectively controlled by osteoblasts and osteoclasts.^5^ In MBD, the mechanism is found mainly to be the imbalance between the markedly increase in osteoclast activity and the inhibition of osteoblast activity.^3^ Improvement of osteoblast activity may both promote the bone formation and prolong the survival time for the MBD patients.^5^

CCN family proteins are involved in many cellular processes including proliferation, differentiation, adhesion, angiogenesis, and survival. The family plays a critical role in bone tissues and contributes to osteoblastogenesis at multiple stages. Among all the members of CCN family, CCN1 can significantly induce osteoblast proliferation.^10^ In the previous study, CCN1 was found to be related to superior progression‐free and overall survival with high concentration.^11^ High level of CCN1 can also reduce the tumor growth and prevent bone destruction in mice.^11^ In our previous research, we found that the quantity and mineralization of osteoblasts could increase markedly after CCN1 stimulation.^12^ Runt‐related transcription factor 2 (Runx2) and *β*‐catenin, the transcription factors of Wnt pathway, were upregulated in osteoblasts after CCN1 stimulation, which revealed their probable involvement in this procedure.^12^ This study showed another potential way to treat MBD. However, the specific mechanism of how CCN1 work to promote the proliferation of osteoblasts is still unclear. As reported, PI3K/AKT signal pathway contributed to the proliferation of preosteoblasts in SLA.^13^, ^14^ Thus, this study aims to identify the signal pathway in CCN1 stimulation on osteoblasts.

## 2 MATERIALS AND METHODS

### 2.1 Patient population

Twenty‐eight myeloma patients were enrolled in this study. The patients came from the Hematology Department of Tianjin General Hospital during October 2017 to August 2018. The clinical diagnostic criterion was based on the International Myeloma Working Group convention.^15^ The baseline characteristics of the patients are shown in Table 1. The two healthy donors were a 61‐year‐old male and a 52‐year‐old female, who had similar age and sex compared with the patients. Ten milliliters of BM samples were obtained from each healthy donors and myeloma patients. This study was approved by the Ethical Committee of the Tianjin Medical University. A written informed consent was obtained from the patients for the publication.

**Table 1:** Baseline characteristics of the patients

Characteristics	Patients (N = 28)
	n/N (%)
Age (years)	
Median (range)	63.5 (45‐82)
Gender	
Male	18 (64.3%)
Female	10 (35.7%)
ISS stage	
I	14 (50.0%)
II	5 (17.9%)
III	9 (32.1%)
Bone disease stage	
0	6 (21.4%)
1	9 (32.1%)
2	8 (28.6%)
3	3 (10.7%)
4	2 (7.1%)

Abbreviation: ISS, International staging system.

### 2.2 Cell culture

**Figure 1:**
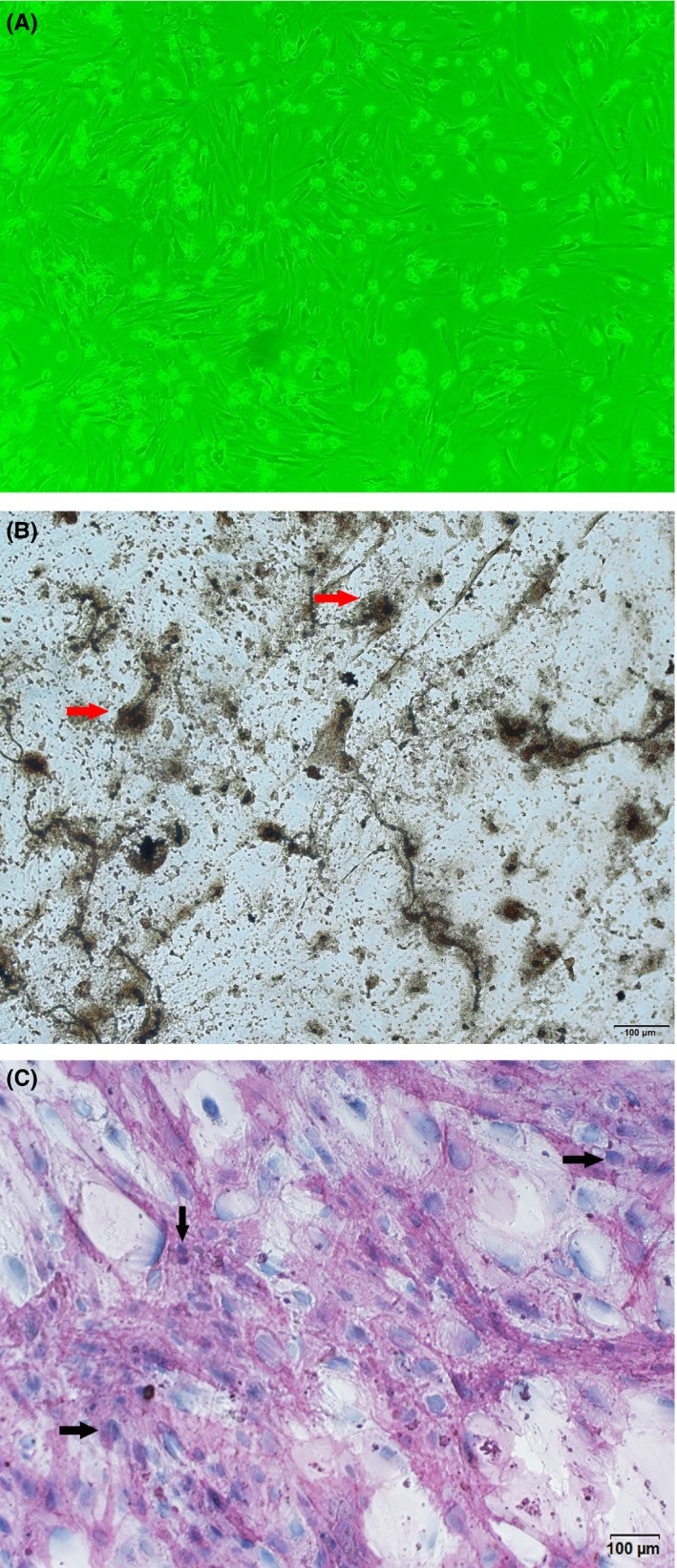
Osteoblasts derived from monocular cells of myeloma patients. A, osteoblasts under microscope, 10 × 10; (B) osteoblasts in Von Kossa staining; (C) osteoblasts in alkaline phosphatase staining

The bone marrow mononuclear cells (BMMNCs) were separated using Ficoll‐Hypaque density sedimentation. The BMMNCs were cultured in Dulbecco's modified Eagle's medium/F12 medium supplemented with 15% fetal bovine serum (Gibco, Darmstadt, Germany), 1 × 10^−7^ mol/L dexamethasone, 0.05 g/L vitamin C, 0.01 mol/L *β*‐sodium glycerophosphate (Sigma), 100 g/mL penicillin (Hyclone), and 100 U/mL streptomycin (Hyclone). Non‐adherent cells were removed the next day, and the media were replaced twice a week. Adherent BMMNCs were cultured at 37°C in an atmosphere containing 5% CO_2_. The OBs were counted and seeded in six‐well plates at a plating density of 2 × 10^5^ cells/mL. The cells were proven by the alkaline phosphatase (ALP) and Von Kossa staining (Figure 1). Trypsin was used to detach three wells of OBs for cell count every second day.

The OBs were divided into two groups in the first part: the control group and CCN1 group (30 ng/L), and divided into three groups in the second part: the control group, CCN1 group (30 ng/L), and TWS119 group (2 μmol/L) (Sigma Company). Before collecting the cells for protein lysis, all the groups were incubated for 72 hours.

### 2.3 Intracellular signaling antibody array kit

Nine samples of myeloma patients and two samples of healthy donors were obtained in this experiment. The experiment was performed strictly following the protocol of the PathScan® Akt Signaling Antibody Array Kit (Chemiluminescent Readout) (Cell Signaling Technology).

### 2.4 Western blot

Cellular lysates were derived from prior study,^16^ proteins resolved by sodium dodecyl sulfate‐polyacrylamide gel electrophoresis and then transferred to nitrocellulose filter membrane (Millipore, MA). Blots were blocked with 5% non‐fat milk for one hour at room temperature, and then incubated with rabbit anti‐human antibodies against p‐AKT (Cell Signal Technology Company, #9188S), AKT (Cell Signal Technology Company, #4691S), p‐GSK3*β* (Cell Signal Technology Company, #5558S), GSK3*β* (Cell Signal Technology Company, #9315S), cyclinD1 (Cell Signal Technology Company, #3741S), GAPDH (Cell Signal Technology Company, #5174S) or *β*‐actin (Cell Signal Technology Company, #8457S) (1:1000) overnight at 4°C. After three washes, blots were incubated for 1 hour with goat anti‐rabbit peroxidase‐conjugated secondary antibody (Beyotime Company, A0277) (1:2500) at room temperature. After three washes the blots were visualized by enhanced chemiluminescence with chemiluminescence system machine (Clinx Company, ChemiScope 6300). All the samples had been tested twice.

### 2.5 Statistics

Results were analyzed with GraphPad Prism (Version 6.0). The data were expressed as mean ± SD. The calculations of the blots’ grey‐scale value were performed by ImageJ. The results were analyzed by performing the Wilcoxon signed‐rank test. *P* < .05 (*) was considered to be statistically significant.

## 3 RESULTS

### 3.1 CCN1 can stimulate the proliferation and differentiation of osteoblasts in MBD patients

Ten samples from MM patients were cultured and divided into control group and CCN1 group to confirm the stimulation of OBs. The OBs were incubated with or without CCN1 (30 ng/L) for 72 hours in vitro and the quantity of OBs were subsequently observed. The OB quantity cultured with CCN1 (30 ng/L) was (3.73 ± 1.16) × 10^5^/mL while the quantity was (2.84 ± 0.31) × 10^5^/mL in control group. There was significant difference between the control group and CCN1 group (*P* = .041).

### 3.2 PI3K/AKT/GSK3β signal pathway was screened out by specific intracellular signaling antibody array

**Figure 2:**
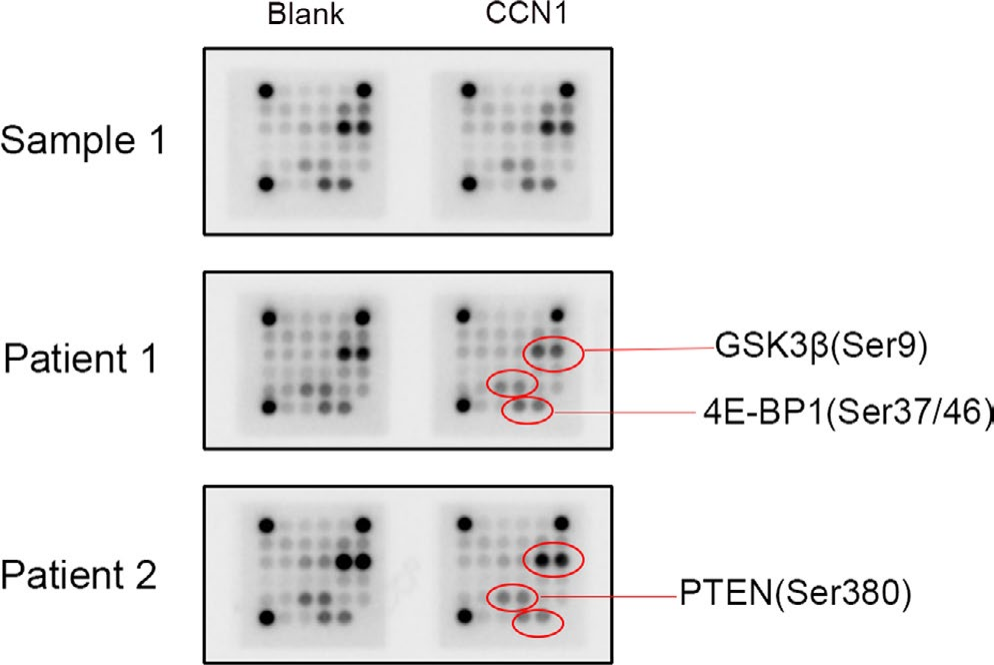
Expression levels of different proteins in osteoblasts changed after co‐cultured with CCN1 for 72 h by AKT signaling antibody array test. Sample 1 is from one of the healthy donors, and all the testing spots on the plate had no obvious change after cultured with CCN1 for 72 h. However, the samples from myeloma bone disease patients (Patient 1 and Patient 2) both had remarkable decrease in the testing spot of GSK3beta, PTEN, and 4E‐BP1 protein after the co‐culture. These results suggested that the CCN1 might have worked directly on these spots of signal pathways

To investigate the possible involvement of PI3K‐AKT signal pathway in the CCN1 stimulating effect on osteoblasts, we first test our osteoblasts samples from both myeloma patients and healthy donors by PI3K‐AKT specific intracellular signaling antibody array. For the two healthy donors, neither of their samples showed any difference between the control group and the CCN1 group. All the testing pots had almost the same degree of expression intensity (Figure 2). However, for myeloma patients, several testing pots had obvious difference between the two groups. GSK3*β*, PTEN, and 4E‐BP1 were the most common ones that had the differences (Figure 2). Among all the nine samples, five of them had decreased expression intensity on GSK3*β* (Ser9) and 4E‐BP1 (Thr37/46) in CCN1 group; four of them had decrease in PTEN (Ser380) in CCN1 group. Three of the patient samples had the same expressions for two groups, just as the healthy donor samples. Besides, two of the patient samples also had a decrease in Erk1/2 (Thr202/Tyr204) in CCN1 group compared with the control group. According to these results, we suppose that PI3K/AKT signal pathway has involvement in the CCN1 stimulation on osteoblasts, especially for the myeloma patients.

### 3.3 Activated PI3K/AKT/GSK3β signal pathway in the osteoblasts was identified by WB after CCN1 stimulation

**Figure 3:**
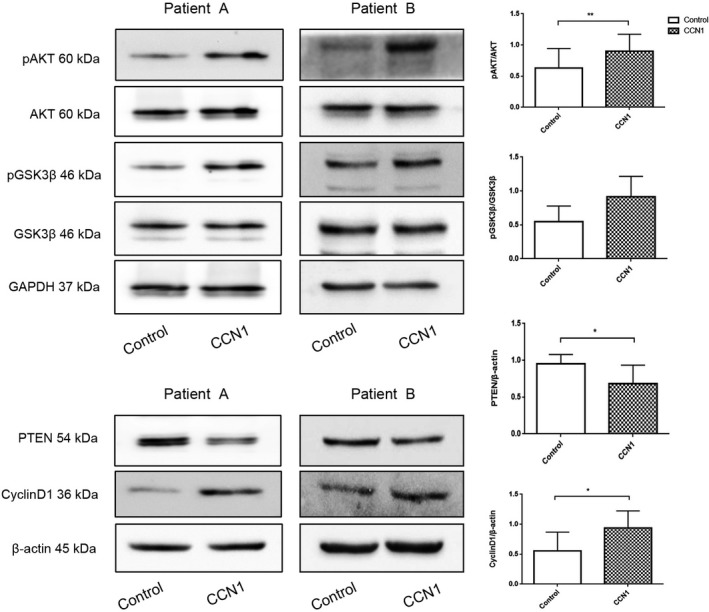
CCN1 had effect on PI3K‐AKT signal pathway in osteoblasts derived from myeloma patients. Control group was cultured only with medium while CCN1 group was cultured with CCN1 at concentration of 30 ng/mL for 72 h (n = 10, eight of them with MBD). GAPDH and *β*‐actin served as loading control. *P < .05; **P < .01

Thus, we took western blot experiments to test the expression levels of PTEN, AKT, p‐AKT, GSK3*β*, p‐GSK3*β*, and cyclinD1 in the osteoblasts from ten myeloma patients. We also put each of the patient samples into two groups: the control group and CCN1 group. The results showed that total AKT and total GSK3*β* almost had no difference in expression level between the two groups while the other four proteins had some significant changes (Figure 3). Comparing to the blank group, some of the samples had increase in p‐AKT, p‐GSK3*β*, and cyclinD1 while they had a decrease in PTEN. However, there were some samples which had no obvious differences or even had opposite trends of differences in these four proteins. To investigate whether the two groups had any significant difference on these protein expressions, we calculated the IOD of the grey shadow of the western blot images and compared them between the two groups. The results showed that CCN1 group had a lower expression for PTEN (*P* = .031) as well as higher expression for p‐AKT/AKT (*P* = .002) and cyclinD1 (*P* = .039) compared with the control group. The total AKT and total GSK3*β* presented no difference in the two groups. The p‐GSK3*β*/GSK3*β* was higher in CCN1 group, but it could not reach a significant difference (Figure 3).

### 3.4 The role of CCN1 on osteoblasts is similar with GSK3 inhibitor TWS119

**Figure 4:**
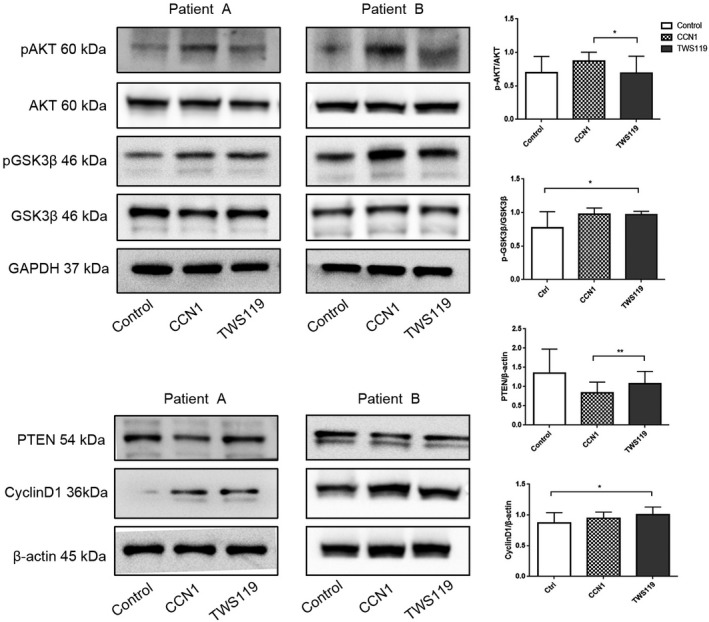
CCN1 and GSK3*β* inhibitor TWS119 had the same effect on decreasing the viability of GSK3*β*. Control group was cultured only with medium, CCN1 group was cultured with CCN1 at concentration of 30 ng/mL and TWS119 group was cultured with TWS119 at concentration of 2 μmol/L. The three groups were all incubated for 72 h. GAPDH and *β*‐actin served as loading control (n = 9, eight of them with MBD). *P < .05; **P < .01

After performing the western blot on these ten samples, we had known that CCN1 did have some influence on the PTEN‐AKT‐ GSK3*β* signal pathway. PTEN expression decreased while the phosphate‐AKT expression increased, thus AKT activity also increased and then inhibited the GSK3*β* activity. This was also confirmed in our experiments, p‐GSK3*β* expression level increased in CCN1 group. But we are still unable to determine to what extent this effect can be achieved, and whether the effects of CCN1 can inhibit GSK3 *β* as GSK3*β*inhibitors. To solve this question, we chose GSK3 inhibitor TWS119 as another control. Eleven patient samples of osteoblasts were collected; each of them was divided into three groups: the control group, CCN1 group, and TWS119 group. After incubation for 72 hours, we performed western blot on them. As the results showed, CCN1 could have almost the same effect on GSK3*β* as the specific inhibitor TWS119 had (Figure 4). The control group and TWS119 group had similar expression level on upstream proteins such as PTEN and p‐AKT. Comparing to CCN1 group, TWS119 group was higher for PTEN (*P* = .001) and lower for p‐AKT/AKT (*P* = .025). For downstream protein cyclinD1, both TWS119 group and CCN1 group had significant increase compared to the control group. However, the CCN1 group had presented a much more complete regulate effect from PTEN all the way to cyclinD1. Thus, these results imply us that CCN1 can stimulate the proliferation and progression of osteoblasts from myeloma patients through PTEN‐AKT‐GSK3*β* signal pathway.

## 4 DISCUSSION

For the past decade, CCN1 has been found to have a positive effect on the proliferation and growth of bone tissue in many bone‐related diseases. For bone fracture, CCN1 serves as an important regulator of bone healing.^17^ In mice experiment, blocking CCN1 would prevent bone fracture from healing.^18^ For arthritis, CCN1 has been shown to stimulate abnormal proliferation and upgrade oncostatin M (OSM) expression in osteoblasts.^19^, ^20^ As for the development of cartilage, CCN1 overexpression promotes chondrocyte maturation and can be induced by WNT/*β*‐CATENIN signaling.^21^ In 2011, Harumi Kawaki et al had shown that Smad and MAPK signaling pathways involved in the positive effect which CCN family had on osteoblasts.^10^ In myeloma field, Sarah K. Johnson et al had reported that CCN1 level is related to the progression and overall survival of multiple myeloma and signaling through *α*_v_ *β*_3_ was required for CCN1 prevention of bone disease.^11^ They found that the blockade of CCN1 interaction with *α*v*β*3 integrin would reduce CCN1‐mediated osteolysis.^11^ At the same time, the blockade would also reduce the CCN1 inhibition effect on tumor growth.^11^ In our previous study, we also analyzed the mRNA expressions of Runx2, *β*‐catenin, and BMP2 in the CCN1 treating osteoblasts. We found that mRNA of *β*‐catenin and Runx2 had significant increase after CCN1 stimulation.^12^ And they are critical transcription factors of early osteoblast differentiation.^22^ In this study, we were trying to focus more on the mechanism of how CCN1 stimulate the proliferation of osteoblasts itself in myeloma patients.

In order to mimic the osteoblasts in MBD patients, we chose the bone marrow mononuclear cells directly from myeloma patients and induced them differentiated into osteoblasts. Thus the samples we used were approximately similar to the osteoblasts in MBD pathologic status. As PI3K/AKT signal pathway plays crucial roles in cell cycle regulations,^23^ we wondered if it also had any involvement in the stimulation effect of CCN1. So we first collected several samples including the healthy donors and the myeloma patients, incubated with medium or CCN1 and then tested by the intracellular signaling antibody array. The results showed that CCN1 would have almost no effect on healthy donors while the myeloma patients had apparent difference. We thought this may because the healthy donors did not have pathologic bones and their osteoblasts were in a physiological balance statue. It seemed that CCN1 would not disturb the regulation of intracellular signal pathway in osteoblasts coming from a physiological normal host. As in the myeloma patients samples, several protein pots including PTEN, GSK3*β*, and 4E‐BP1 in the array had been observed have involvements.

Phosphatase and tensin homolog deleted on chromosome ten (PTEN) was originally identified as a tumor suppressor and can inhibit the activation of the phosphoinositide 3‐kinase (PI3K)–AKT pathway, which is important in cell growth and survival.^16^, ^24^, ^25^ Thus, inactivating PTEN can activate PI3K‐AKT in turn; PTEN works as a negative regulator for PI3K‐AKT.^24^ PTEN can also decrease cyclin D1 expression, downregulate the protein stability, and inhibit its nuclear localization.^26^, ^27^ Glycogen synthase kinase‐3 (GSK3) has been found involved in many diverse pathways related to cell activities such as metabolism, differentiation, and apoptosis.^28^, ^29^ GSK3*β* is one of the two isoforms of GSK3, and can be phosphorylated by all three isoforms of AKT.^30^ PI3K/AKT activation can lead to GSK3 inactivation and AKT is the primary kinase responsible for phosphorylation of GSK3 at S9 in vivo.^23^, ^31^, ^32^ Cyclin D1 protein level is also regulated by GSK‐3. AKT can directly phosphorylate and inactivate GSK‐3, which will then inhibit degradation of cyclin D1 induced by GSK‐3.^23^

4E‐binding protein 1 (4E‐BP1) has tumor suppression effect by blocking mRNA translation and proliferation.^33^ This effect is realized by binding with eIF4E and inhibiting its activity, which can lead to decrease in overall translation rate.^33^ Thus 4E‐BP1 is kind of negative regulator for cell cycle progression, cell growth, and cell proliferation. In our experiments, 4E‐BP1 had presented an obvious decrease in osteoblasts which were co‐cultured with CCN1. This result may suggest that the 4E‐BP1 is also involved in the CCN1 stimulation effect on osteoblasts.

Comparing to the control group, PTEN level decreased in CCN1 group while p‐AKT/AKT, p‐GSK3*β*/GSK3*β*, and cyclin D1 levels were all increased. These results can explain how CCN1 stimulate the osteoblasts through the PI3K‐AKT signal pathway. CCN1 first inhibited PTEN expression in the upstream, which would lead to the activation of AKT; thus phosphate‐AKT/AKT level increased. The activation of AKT then inhibited the GSK3*β* activity; more GSK3*β* were phosphated and inactivated, which could activate cyclinD1 in the downstream. Because of the inhibition of PTEN and the activation of AKT, cyclin D1 also got activated and its expression level increased. The result then led to the increase in proliferation and growth in osteoblasts. At the second time of western blots, we chose TWS119 as another group because there was no available agonist of GSK3*β*. The results showed that CCN1 could have as same degree of inhibition as the TWS119 had on GSK3*β*, which meant that CCN1 had a very strong effect on GSK3*β*. This also proved that CCN1 do have stimulation on the osteoblasts of myeloma patients through PI3K/AKT‐GSK3*β* pathway. Because PTEN, 4E‐BP1, and PI3K‐AKT are popular protein targets involved in diverse of cancers, there might be concerns that whether CCN1 would increase the possibility of myeloma progression. But according to the research Sarah K. Johnson et al have made, CCN1 can even prevent the progression MM,^11^ thus the concern is not a problem. Besides, as CCN1 showed almost no effect on the osteoblasts from healthy donors, we think that it may be a potential way to treat with MBD with much less concern about adverse effect.

## 5 CONCLUSION

CCN1 can stimulate osteoblasts in myeloma bone disease through PTEN‐AKT‐GSK3*β*‐cyclinD1 pathway while it has no additional effect on osteoblasts in healthy beings. Thus CCN1 has the potential to be another method to treat myeloma bone disease.

## CONFLICTS OF INTEREST

All authors report no conflicts of interest.
